# Rules for House Drainage

**Published:** 1881-05

**Authors:** 


### RULES FOR HOUSE DRAINAGE.

The Secretary to the "Office of Works" in London has issued the following excellent sanitary rules in constructing buildings:

"1. All water-closets and urinals shall be constructed so that one wall, at least, of such closets and urinals shall be an outer wall of the building.

"2. All soil-pipes shall be carried outside the building, and ventilated by means of pipes leading the foul gases above the highest point of the building, such pipes to be carried to points removed from chimney-stacks.

"3. Seperate cisterns shall be constructed for the water-closets, and for the general purposes of the building. No tap or 'draw-off' shall be affixed to any pipe communicating with a cistern supplying a water-closet or urinal.

"4. All waste-pipes and overflow-pipes of cisterns shall terminate in the open air, and be cut off from all direct communication with drains.

"5. Great attention shall be paid to insuring thorough ventilation in all rooms. Rooms so high that their ceilings shall be more than two feet from the top of the windows, corridors, staircases, and other open spaces, shall be specially ventilated so as to prevent the accumulation of stagnant air.

"6. All main drains should, where practicable, be formed outside the building. In the event of its being necessary to carry a main drain underneath a building, it must be trapped immediately outside the main wall, and a ventilating shaft must be carried from that point to the highest part of the roof, as under Rule 2."—*The Sanitarian.*

